# Who Panics When They Think of Work?

**DOI:** 10.3390/ijerph22020160

**Published:** 2025-01-25

**Authors:** Beate Muschalla

**Affiliations:** Psychotherapy and Diagnostics, Technische Universität Braunschweig, 38106 Braunschweig, Germany; b.muschalla@tu-braunschweig.de

**Keywords:** work anxiety, work phobic anxiety, workplace phobia, coping, work ability, life satisfaction, values, unemployment

## Abstract

Work anxiety is a specific mental health problem that is often associated with sick leave and negative work perception. Until now, there has been hardly any evidence on the more general psychological characteristics of work-anxious people, i.e., in terms of life values, life satisfaction, work coping, and activity level. Learning more about these cognitive and behavioral characteristics is, however, of interest for preventive action. This present study investigates these characteristics in people with and without mental health problems and with and without work anxiety comparatively. A representative sample of 2131 persons from the German general population of working age (18–70 years old, considering that many people wish to continue work after official retirement at age 67) were investigated via an interview and self-rating questionnaire. People with work anxiety have more work participation problems in terms of unemployment (8–20% of work-anxious were presently unemployed vs. 3–10% of those without work anxiety) and sick leave (1.6–7.0 weeks in the past 12 months vs. 1.2–4.8 weeks) compared with people without work anxiety. People with work anxiety show specific patterns of negative ratings of work-associated life satisfaction (work and colleagues) and place increased value on power and the need for control. In non-work-related life domains (neighbors, friends, and environment), there are smaller satisfaction differences between people with and without work anxiety. In conclusion, work anxiety is a specific mental health problem that is associated with specific work-related participation and life satisfaction problems (partly different from people with general mental health problems). Work anxiety must be specifically assessed in order to enable preventive or interventive action.

## 1. Introduction

Work anxiety is anxiety related to the workplace. According to the psychopathological concept of anxieties [1], work anxiety can occur as work-related anxiety arousal, anticipatory anxiety thoughts, and panic when being in certain anxiety-provoking situations at work (e.g., specific job tasks, social situations, achievement demands, or uncertainties at work), or even when thinking of these or the workplace itself.

In contrast to general anxiety, work anxiety is specifically associated with the workplace. Work anxiety can, if undetected, result in avoidance behavior concerning the workplace and, therefore, result in (long-term) sick leave [2,3,4,5,6]. Work anxiety appears partly independent from other mental health problems: work anxiety may occur as a standalone mental health problem (without any other mental comorbidities). Due to its specific content-relatedness, work anxiety needs a specific diagnosis and treatment [2,5].

One problem regarding work anxiety is that it is often overseen and not labeled concretely, but rather covered by unspecific terms like stress, burnout, or dissatisfaction with the workplace [6]. The second problem is that work-related avoidance often results in sick leave certification because being sick-listed is the only legal way to stay away from the workplace [2,4]. Once the work-anxious person becomes sick-listed, it is too late for preventive action regarding their working ability. If already sick-listed, it is more complicated to return to work in case of work anxiety because the anxiety–avoidance is directed toward the workplace. Thus, avoidance and being away from work (on sick leave) reduces anxiety in the short term, but it decreases the probability of returning to work and the ability to work in the long run [5].

A good strategy might be to detect work anxiety early. This can be achieved by being aware of specific patterns of cognitive and behavioral signs that can often be found in work-anxious people, e.g., a negative work perception [6]. These may have a certain indicative value, i.e., they might signal a potential work anxiety problem.

Therefore, a more specific characteristic of people who are at risk of work anxiety is needed. The question is which characteristics are often found in people with work anxiety but not so often in healthy people, or people with mental health problems. The large nationally representative survey described herein of people of employable age aims to increase knowledge about which cognitive and behavioral characteristics often accompany work anxiety.

### 1.1. Early Detection of Work Anxiety Is Needed

Little is known and conducted with respect to the prevention and early detection of work anxiety in vocational settings. In organizations, it would be useful to bear the concept of work anxiety in mind when it comes to the negative effects at work, conflict, dissatisfaction, deviance, or workplace absence on employees [3,7,8].

Although stress perception is different between individuals (according to the transactional stress model [9]), there are some basic stressors that may trigger irritation, stress, and anxiety in most people, i.e., uncertainty, high demands, and, at the same time, few resources to cope with the demands (demand–control model, [10]), or job tasks that do not fit the person’s capacity profile (person–job fit [11,12]). Other triggers of an individual’s work anxiety could be specific work situations, such as a computer-heavy workplace; special work tasks; working hours; work locations; or work environments, such as night shifts or a workplace with increased demands on personal responsibility, health endangerment, or social conflicts, and mobbing [3,8,9,10,11,12,13,14,15,16,17]. It is empirically known that work-anxious people describe their work situation with a more negative affect as compared to people without work anxiety [6].

Work anxiety and its treatment have been investigated broadly in clinical settings in patients with different chronic illnesses [2,5,13,18]. However, until now, not much has been known about work anxiety and its correlates in the general working population [19,20], as opposed to specific work settings [21,22]. Until now, there has been only one previous representative study carried out in 2019, which suggests that about 7% of people of German working age have work anxiety problems [20] according to the Workplace Phobia Scale [23,24]. Similarly, other empirical studies in different working samples have reported a rate of about 3–5% of severe work anxiety [19,21]. In primary care patients with chronic mental illness, the rate of patients with work-phobic anxiety is about 10%, and in inpatient settings, this reaches 17% [24,25]. These are relevant numbers, given the potential negative consequences on the affected employee (long-term sick leave and unemployment) and the organization (productivity loss).

### 1.2. Work Anxiety Core Phenomenology

In clinical research, it is known that there are different phenotypes of work anxiety, similar to the general psychopathology of anxiety [1]. The identified work anxieties are as follows: work-related situational anxiety, work-related social anxiety, work-related worrying, work-related hypochondriac or functional anxiety, and workplace phobia [4,13,14,15,24,26]. However, are there *general core aspects* that characterize work anxiety? Yes, indeed: all work anxiety phenotypes come along with anxiety or even panic when being confronted with or when thinking of the workplace or specific triggers or situations at work [13,14,24,26]. Thus, work anxiety usually has an *anticipatory aspect*: work anxiety is not only present when the person is at work but also arises when thinking of the workplace, an upcoming working situation, or a working day. Anticipatory work anxiety may include anxiety toward the workplace itself, a defined place, a certain task, or a situation at work [6,13,14,15,16,17,26].

A second common core aspect of work anxiety is *physiological arousal, tension, anxiety, or even panic* when imagining or approaching the workplace [24,26].

According to the core phenomenology of anticipatory work-related physiological tension, work anxiety can be explored by asking whether the person feels panic when thinking about the workplace or spending a whole working day there.

### 1.3. Objectives

Evidence until now shows that work anxiety moderately correlates with mental health aspects and different potential work situation triggers: work anxiety has been found to occur with other mental health problems such as embitterment, increased sick leave, and (work) capacity impairments [20]. There are also correlations of work anxiety with general mental health burdens [21,22,26,27,28] and event-related stress symptoms [11]. Work-anxious people report different work-related stressors [6].

However, there has been, until now, hardly any evidence on more general psychological characteristics of work-anxious people, i.e., in terms of life values [29,30], life satisfaction [31], work coping [5], and activity level [32]. To know more about potentially typical cognitive and behavioral characteristics would, however, be of interest for preventive action: do employees prone to work anxiety (and thus candidates for potential work avoidance and sick leave) have specific life perceptions or behavioral characteristics that could receive attention in work ability prevention? Are there cognitive or behavioral characteristics that can be often observed in those who are work anxious but less often in healthy people or those with general mental health problems?

This present representative study aims to fill this gap of knowledge by investigating these characteristics in people with and without mental health problems and with and without work anxiety comparatively.

Research questions:

1. Which sociodemographic characteristics are typical in people with work anxiety (A), compared to healthy people (NN), people with mental health problems (M), and people with work anxiety and mental health problems (AM)?
2. As earlier research shows, work anxiety is often associated with work ability problems [4,24]. Therefore, the hypothesis is that people with work anxiety (A, AM) have longer sick leave and longer unemployment times than people without work anxiety (NN, M).
3. As earlier research has shown, people with work anxiety have a more negative work perception [6]. Therefore, the hypothesis concerning cognitive characteristics—in terms of life values and life satisfaction—is that people with work anxiety (A, AM) report less satisfaction in work-related life aspects as compared to healthy people (NN) and people with mental health problems (M).
4. As earlier research showed, people with work anxiety have work coping problems [5]. Therefore, the hypothesis concerning behavioral characteristics is that people with work anxiety (A, AM) report lower work coping as compared to healthy people (NN) or people with mental health problems but without work anxiety (M).

## 2. Materials and Methods

### 2.1. Procedure

A large representative cohort of national inhabitants of all age groups was investigated in 2022 [33]. The investigation was carried out by a German professional representative study company (USUMA GmbH). The representative sampling was based on a nationwide division of the populated area of Germany into sample areas. An overlap-free spatial definition of sample areas was assured. Afterward, areas were chosen. Within these areas, a random selection of households was performed. For person selection, an administrative person for the respective area must identify all people in the selected household and select a target person for the interview. This selection of the target person was performed via a predetermined random procedure according to the internationally used “Kish-Selection-Grid” [34]. The Kish grid is a means for random choice of survey respondents in households. The Kish grid aims to avoid bias by assigning age-based numbers to each household member. The most important aim is to assign an equal selection probability for each possible survey participant.

The representative sampling procedure was independent of the interviewer. In the face-to-face interview, which was carried out by a trained interviewer, the randomly chosen participants were asked to provide basic socio-demographic and work-related data (Table 1). After the interview, participants completed a self-rating questionnaire beginning with the core question of whether they suffered from chronic mental health problems (see Section 2.3.) and work-related anticipatory panic-link anxiety (2.4. WPS), followed by psychometric questionnaires on their subjective job coping capacities (2.5. JoCoRi), their recreational daily activities (2.6. iRADL), life values (2.7. SVS), and satisfaction with different life domains (2.8. DLB).

### 2.2. Participants

A total of 2522 people of all age groups (16–96 years) from the general population were randomly selected and investigated. Only people with severe barriers to understanding language or severe disability (i.e., inability to answer questions) were excluded. Retired or unemployed people were included.

A total of 2131 participants of employable age (18–70 years) could be included in the statistical analysis with sufficient data. Half of the investigated were female (49.5%), and the age was, on average, 45.54 years (Table 1). Two-thirds had a religious affiliation, and two-thirds lived together with a partner. A total of 4.6% were presently unemployed.

### 2.3. Chronic Mental Health Problem

Participants were asked whether they suffered from chronic mental health problems with accompanying impairments. The definition of mental health problems in this investigation was according to the definition of common mental disorders (anxiety, depression, and adjustment disorders). Chronic mental health problems are defined by specific symptomatology with suffering and impairment in daily life activities and coping with stress or life challenges [25,31,35]. The majority of patients who are in treatment with mental health problems are chronic (80%); mental disorders are lifetime disorders by nature [25,35,36].

In the present representative study, chronic mental health problems were assessed with the following content-valid questions: “Do you regularly have—now and earlier—complaints such as anxiety, mood- or interactional problems, which lead to impairments in your daily life routines? Have you been in treatment because of these problems or have others suggested that you should go into treatment (by physicians or psychologists)?” In case the participant answered this with “yes” (answer categories: yes or no), he or she was classified as person with mental health problems and grouped accordingly for analysis (Table 1).

Chronic mental health problems were assessed in this way in various other studies [20,25,35,37]. This way of asking for chronic mental health problems has been proven content-valid and comprehensible and has also been validated with a standardized interview: in an earlier validation study, 307 patients in primary care services reported chronic mental health problems according to the above-mentioned definition and assessment questions in a self-rating questionnaire. All of them fulfilled criteria of any common mental disorder according to the structured diagnostic MINI interview [25,38]. The self-rating and the MINI interview were performed independently.

### 2.4. Work-Related Anticipatory Panic-like Anxiety (Workplace Phobia Scale—WPS)

To assess anticipatory workplace anxiety, we used one core item of the workplace phobia scale (WPS). The WPS [23,24] is a 13-item self-rating scale for measuring work-phobic anxiety, i.e., anxiety with work-related panic and work-related avoidance behavior. The WPS’s psychometric properties were tested using a psychosomatic inpatient sample, providing a split-half reliability of 0.97 and Cronbach’s alpha of 0.96. All items of the WPS items were rated on a Likert scale, ranging from 0 = no agreement to 4 = full agreement. The WPS has been validated with structured diagnostic interviews as criteria [24]. Items of the WPS are given to the participants with the information that the questionnaire asks for “behavior, thoughts, and feelings that can occur in relation to the workplace”. 

In this present investigation, the following core item of the WPS was used for assessing the degree of work-related panic-like anxiety: “When imagining a complete working day at this workplace. I get feelings of panic”. This item is most content valid for the concept of work-related global panic-like anxiety as described above (1.2 work anxiety core phenomenology). It also has a high item-scale correlation (r = 0.843 [23]), which indicates that the item is a very good representative of the whole workplace phobia scale. Those with a rating score of 3 or 4 on this item were grouped as “work-anxious”.

### 2.5. Job Coping: Short Job Coping Scale (JoCoRi)

Participants were asked to give a short rating on their perceived work coping perception on seven coping items (Cronbach’s alpha 0.822) using an evaluated work coping scale (JoCoRi [5]). The instruction above the short work coping questionnaire was: “Please imagine being at your workplace right now. How could you do the following things?” This technique is called cognitive rehearsal and is used in cognitive behavioral interventions (e.g., [39]). This technic means exposure in sensu because the person is required to imagine being at the workplace. Each item is rated from 0 = not able to do this to 4 = best coping ability for doing this. For detailed data analysis, mean scores of each of the seven work coping items were used.

### 2.6. Activities: Assessment of Intentional Activities (iRADL)

Participants filled in the iRADL scale (intentional recreational activities of daily living), a short version of the RADL-ICF-scale [32,40]. The scale covers 12 leisure time and recreational activities: “social encounters”, “television and internet”, “relaxation and silence”, “cooking and eating”, “excursions, traveling, and ventures”, “listening to and making music”, “hobbies”, “wellness and self-care”, “reading and literature”, “sports and exercise”, “activities in nature”, and “enjoying cultural activities”. Participants indicate with yes and no for each item: “I deliberately and regularly do this so that I feel better”. Each item is rated from 1 = I never do this, 2 = I do this seldomly, 3 = I do this often, to 4 = I do this regularly in order to feel better. For data analysis, the percentages were included of those people who perform the respective 12 activities regularly with the intention of feeling better.

### 2.7. Life Values: Short Version of Schwartz’s Value Survey (SVS)

This short value scale is a shortened version of Schwartz’s Value Survey (SVS [41,42]). The original scale (i.e., the long version) includes 57 value items representing ten motivationally distinct values. The Short Schwartz’s Value Survey provides insight into ten broad values. The scale contains four subdimensions. Cronbach’s alpha of the whole scale in this present investigation was 0.837; for the subdimensions power and achievement, it was 0.844; for benevolence and universalism, it was 0.840; for tradition, conformity, and security, it was 0.747; and for hedonism, stimulation, and self-direction, it was 0.737. These Cronbach’s speak for the validity of the scales in reflecting different contents and, therefore, the items being moderately correlated but not redundant. For data analysis, mean scores of each of the ten value dimensions were used. Each item is rated from 1 = not at all important to 4 = very important.

### 2.8. Life Domains: Differential Life Burden or Satisfaction Scale (DLB)

The differential life burden scale [31] asks for general satisfaction with 17 life domains: “partnership”, “sexuality”, “children”, “parents”, “friends”, “neighbors”, “colleagues”, “work”, “leisure time”, “health”, “finances”, “housing”, “environment”, “homeland”, “politics”, “future”, and “life review”. Each item is rated on a bipolar 6-point scale from 1 = very negative to 6 = very positive. The scale was validated in a general population sample of persons aged 20–65 [31] and also used in clinical samples [43]. Cronbach’s alpha of the whole scale in this present investigation was 0.887. This speaks to the validity of the scale in reflecting different life domains, assuming an underlying general satisfaction level in the sense of g factor. For data analysis, mean scores of each of the 17 life domains were used.

### 2.9. Statistical Analysis

The assessment questions for work anxiety (2.4.) and chronic mental health problems (2.3.) were used for classifying the participants into the four groups. To test for differences between the four groups—healthy persons (NN), participants with work anxiety (A), participants with mental health problems (M), and participants with both work anxiety and mental health problems (AM)—we conducted analyses of variance [44] with Bonferroni-corrected post hoc tests or Chi^2^ tests for differences in frequency distributions (for categorical variables). Although data were not fully normally distributed and homogenous in variance, ANOVA was used as it is robust against such violations of preconditions. Significance level for statistical testing was set to *p* < 0.05.

## 3. Results

### 3.1. Socio-Demographics and Work Characteristics

Fundamentally, there were expected differences between people with and without mental health problems: people with mental health problems (M, AM) were more often female (62.8% and 66.0%) compared to the groups without general mental health problems (NN, A). They also had a longer duration of sick leave in the past 12 months (on average, 4.73–7.04 weeks, compared to 1.16–1.57 weeks in NN, A, Table 1).

Unlike the mental health problem groups (AM and M), there was no overrepresentation of women in the work-anxious groups, but rather an equal distribution of male and female gender. Religious affiliation and partner status were similar in terms of participants with work anxiety (A) and healthy participants (NN). People with work anxiety had, on average, more unemployment periods during their lives compared to people without mental health problems. The duration of sick leave was not significantly increased in the work-anxious population in comparison to the healthy population, but more of the work-anxious were presently unemployed (A: 7.9% in contrast to NN: 3.2%, Table 1).

When it comes to a combination of work anxiety and mental health problems (AM, Table 1), work participation impairments are intensively increased: those with both work anxiety and mental health problems were most often unemployed (2.76 times during lifetime, 20% at present), had the longest sick leave duration (7.04 weeks), and had the lowest monthly income.

### 3.2. Life Satisfaction and Life Values

Those without general mental health problems (NN and A) reported the greatest life satisfaction over all life domains and significantly higher satisfaction than those with mental health problems, especially those with comorbid problems (M and AM, Table 2). Combined general mental health problems and additional work anxiety (AM) were associated with the lowest satisfaction in all life domains.

Those with work anxiety (A) were similarly satisfied as the healthy participants (NN) in the non-work life dimensions. Work-anxious participants reported reduced satisfaction for the specific life domains “work” and “colleagues”.

People with mental health problems (M AM) reported exceptionally less satisfaction concerning “health” compared to healthy (NN) and work-anxious participants (A).

All groups reported a similar ranking of several life values (Table 3), with the highest agreement for benevolence, universalism, self-direction, and stability (means > 4), and comparably lower importance for power, achievement, and stimulation (means < 4). Healthy people reported stronger agreements with nearly all life values.

Interestingly, work-anxious participants valued power comparably more than the other three groups. In the majority of the other life values, the work-anxious participants reported similar levels compared to the healthy participants (NN).

### 3.3. Job Coping

In job coping (Table 4), work-anxious (A) and those with mental health problems (M, AM) report similarly low levels, significantly lower than the healthy (NN). This signals that work anxiety is specifically associated with low job coping (even if not combined with mental health problems). In addition, when work anxiety and general mental health problems occur combined (AM), job cooping is even worse.

### 3.4. Intentional Recreative Activities

Work-anxious people more often use TV and the internet as recreational activities in order to feel better (A: 30%, 40%, Table 5) than healthy people or those with general mental health problems (NN: 17.8%, M: 25.7%). Work-anxious people partly use more relaxation and silence (AM: 24%) and engage in more hobbies (A, AM: 16% versus NN: 8.4% or M: 9.6%).

People with combined mental health and work anxiety problems are the least likely to use social encounters for recreational distortion (AM: 18% versus M, NN, A: 23–28%).

## 4. Discussion

This representative general population investigation in people with specific work anxiety compared to healthy people or people with mental health problems shows some special characteristics of work anxiety, which distinguish the phenomenon from general mental health problems or from a healthy status:

Firstly, concerning socio-demographic characteristics, work anxiety occurs with similar frequencies in men and women in the general population. In contrast, general mental health problems are known to be overrepresented in women [45]. This is in line with findings from another general population sample, in which work anxiety occurred in women and men similarly [20]: in participants with moderate or high work anxiety as measured with the Workplace Phobia Scale, 50–54% were female.

Secondly, data from the present representative investigation show that people with work anxiety have more frequent and more severe work participation problems in terms of sick leave duration, present unemployment, or times of unemployment during life. People with work anxiety perceive reduced work coping, similar to people with general mental health problems. Work anxiety, in addition to mental health problems, even comes along with increased work coping problems. These findings are in line with clinical research in patients with chronic illness and work anxiety, which also shows the narrow connectedness of specific work ability problems with specific work anxiety [24]: in a sample of 230 patients with mental disorders, 36 patients with workplace phobic anxiety had been on sick leave for 24 weeks in the past 12 months compared to 11–15 weeks in patients without workplace phobic anxiety but other mental disorders [24]. A total of 70–77% of the 36 work-anxious were on sick leave compared to 33–36% of those without work anxiety. Therefore, the so-called “hard data” on present and past work ability and employment status and problems may function as relevant indicators of future potential work anxiety and work ability problems.

Thirdly, concerning work perception, work-anxious people have a specific perception of work-associated domains of life: they perceive domains of work and colleagues more negatively than the healthy, whereas there is a similar level of satisfaction in work-anxious and healthy in other life domains (neighbors, leisure time, health, future, and life review). The work-anxious people value “power” more than the others (healthy people or those with general mental health problems). In sum, work-anxious people, therefore, seem to have a more problematic view of their work setting and, at the same time, a stronger will to control in domains of achievement. A specific negative perception of work by people with work anxiety was also found in another study [6] that compared 148 psychosomatic patients and 8015 general population controls: patients with work anxiety described their workplace significantly more negatively than patients without work anxiety and employees in the general population, and there were no differences in workplace descriptions between psychosomatic patients without work anxiety and the general population sample. This shows that work anxiety and not general mental health problems as such are specifically related to negative work perception.

Fourthly, concerning job coping, people with work anxiety reported lower coping compared to healthy people. Therefore, even in the case of “work-anxiety only” without general mental health problems, this status already comes along with reduced job coping and becomes even more severe in the case of additional mental health problems. In a therapy study for patients with multiple somatic and additional work anxiety problems, participants reported (reduced) job coping, similar to the work-anxious representative study participants, i.e., job coping means about 2.2–2.5 on a scale from 0 to 4 [2].

### 4.1. Implications for Practice

Work anxiety is specifically associated with work ability problems. For an overview of if or how often work anxiety might be a problem in an organization, it could be useful to integrate one question on work anxiety in employee surveys. In case the surveys are anonymous, of free will, and already include mental health items (besides work perception and environmental aspects), asking one question on work anxiety should not be a problem. The employer could conduct general surveys on work satisfaction and health (including one item on work anxiety) without asking for individual data. Anonymous aggregated data over the whole organization can give a sign as to whether work anxiety is reported rather rarely or frequently in comparison to the known general epidemiology (3–7% work anxiety in the general population [19,20,21]), and thus whether organizational structural aspects should be checked for potential work anxiety stimulation potential. As concerns help individual employees, mental stress could be recorded anonymously by external providers (e.g., company physicians, psychologists, or counseling services). The employer would then only receive aggregated data. Employees could report their mental health problems on a voluntary basis, with the employer ensuring confidentiality and providing support services. Employers could provide training for handling mental health-related work ability issues among managers and employees. These means could help identify problems at an early stage or even reduce them without the need for formal collection of mental health-related individual data.

A potential risk aspect of collecting mental health information in organizations is the discrimination of mental health problems and work anxiety at work, e.g., in recruitment processes, pay raises, promotions, or contract renewal. People with mental health problems may be disadvantaged. Furthermore, colleagues and supervisors are typically not physicians or psychotherapists and thus are not allowed or in a position to diagnose mental health symptoms.

However, even if there is no communication of diagnosis or mental health data in the organization, colleagues and supervisors can regularly *observe mental health-related and work ability problems* in their direct work environment. For example, a colleague with specific anxiety toward a special work task will avoid this task, which may lead to delays, failures, or missed deadlines. This can finally result in the employee’s sick leave or job loss. Therefore, supervisors and teams have to cope with mental health-related work ability problems in concrete work settings. Effectively coping with work-related mental health problems can be achieved by focusing on capacities (instead of mental health symptom diagnosis) according to the internationally evaluated ICF-based psychosocial capacity concept [46,47]. The behavioral capacities of employees can be trained (e.g., via time-, stress-, or decision management coaching). Communication and work duties given by employers should be clear and regular (to avoid overload and irritation). Work duties should be selected for each employee in order to fit the capacity profile of the specific employee. Furthermore, a self-help guide is available for employees and employers [48], based on contents that have been used in an evaluated training against work anxieties [2,5].

In sum, the core idea for organizational practice is to reduce work problems and potential work anxiety by following the person–job fit model [11,12,49]: if the job demands fit the capacities of the employee, the work can be completed well, and the risk of work anxiety can be reduced.

### 4.2. Strengths and Limitations of the Study

The major limitation of this study is the cross-sectional design and self-rating method. However, although there is no diagnosis of mental health problems and work anxiety from observation and clinical interviews, specifically asked self-report data have been proven to be valid in other studies [24]. Mental health problems and work anxiety were assessed with specific and earlier evaluated questions [24] in the present study so that content validity can be expected to be high.

This present study is of descriptive and explorative character since it is the first representative survey on the personal characteristic correlates of work anxiety. Future research could address specific interaction and correlation patterns between work anxiety and personal and environmental characteristics.

Another aspect for further research is the prevalence of work anxiety in different occupational settings. In longitudinal research, the question is under which risk- or protective conditions work anxiety leads to sick leave.

A strength of this investigation is the large and representative sample, which includes all age- and socio-economic groups, as well as the personal interviews for a valid assessment of the sociodemographic data. The large heterogenous sample allows a comparison of all relevant groups (with and without work anxiety). The data add knowledge to the existing research: until now, there were findings of clinical differential diagnostic and the treatment of work anxiety, clinical correlates, and workplace factor correlates (e.g., [5,6,13,18,19,21]). With the present study, we gained additional validation information related to the work anxiety concept: for the first time, general psychological person characteristics were investigated within a national representative sample concerning their associations with work anxiety. With this additional knowledge, the earlier detection of work anxiety problems may become easier.

## 5. Conclusions

This is the first study that investigated life satisfaction aspects differentially in people with and without work anxiety. The representative data show that people with work anxiety do not only have more work participation problems in terms of unemployment and sick leave than others. People with work anxiety also show specific patterns of negative ratings on work-associated life satisfaction aspects (work and colleagues) and increased valuing of power and the need for control. In non-work-related life domains, there are fewer satisfaction differences between people with and without work anxiety.

The findings support previous evidence that shows that work anxiety is a specific and potentially additional health problem that comes along with even more severe work participation and life satisfaction problems (compared to people with general mental health problems only) [6,24].

For occupational health practice and further research, this indicates that the awareness and prevention of work anxiety [48] can be more useful than asking for general mental health symptoms. Work anxiety comes along with special add-on problems (job coping problems, sick leave, unemployment, and work dissatisfaction), which are not found to similar degrees in people with general mental health problems.

People with work anxiety reflect on their work-associated aspects in a specific, negatively biased manner, whereas other life domains are not necessarily negatively affected.

## Figures and Tables

**Table 1:** Characteristics of people of employable age (18–70) without any mental health problems (NN), with mental health problems (M), with work anxiety (A), and both mental health and work anxiety problems (AM) (N = 2131).

	Without Mental Health Problems, Without Work Anxiety (N = 1699) NN	Work Anxiety (N = 191) A	Mental Health Problems (N = 191) M	Work Anxiety and Mental Health Problems (N = 50) AM	All (N = 2131)	Statistics: Overall Differences Between Groups: ANOVA for Mean Scores or Chi^2^ in Frequencies. Significant Differences of Pairwise Comparisons from Bonferroni-Corrected Post Hoc Tests for Mean Scores
Age in years	45.26 (14.21)	47.08 (15.14)	46.04 (14.16)	47.30 (14.29)	45.54 (14.21)	*p* = 0.277, Eta^2^ = 0.002
Gender female %	47.7%	48.2%	62.8%	66.0%	49.5%	*p* < 0.001
Monthly income in EUR	2173.16 (1000.17)	2107.22 (1064.45)	1956.59 (1025.45)	1619.85 (955.36)	2135.44 (1011.66)	*p* < 0.001, Eta^2^ = 0.010 NNvsM 0.031, NNvsAM 0.001, MvaMA 0.018
Duration of sick leave in the past 12 months in weeks	1.16 (3.16)	1.57 (4.48)	4.73 (9.44)	7.04 (12.55)	1.66 (4.81)	*p* < 0.001, Eta^2^ = 0.075 NNvsAM < 0.001, AvsM < 0.001, AvsAM < 0.001, MvsAM 0.010
How often have you been unemployed in your life until now?	0.93 (1.50)	1.28 (1.61)	1.78 (1.79)	2.76 (4.82)	1.08 (1.73)	*p* < 0.001, Eta^2^ = 0.044 NNvsA 0.046, NNvsAM 0.001, AvsM 0.024, AvsAM 0.001, MvsAM 0.002
Presently unemployed	3.2%	7.9%	9.9%	20.0%	4.6%	*p* < 0.001
Religious affiliation	65.7%	65.6%	72.5%	50.0%	66.0%	*p* = 0.025
Living together with partner	67.9%	64.9%	56.9%	57.1%	66.4%	*p* = 0.010

Note: *p*-values and Eta^2^ for overall group comparison are reported in the first line of the Statistics column. In case of significant overall differences in ANOVA, *p*-values of pairwise Bonferroni-corrected post hoc test are shown for continuous variables, e.g., NNvsM 0.031 means there is a significant difference with *p* = 0.031 between the group without mental health or work anxiety problems (NN) and those with mental health problems (M). In case of categorical variables, overall *p*-values are reported, e.g., female gender (*p* < 0.001) occurs with different relative frequencies in the four groups).

**Table 2:** Satisfaction with different life domains (Differential Life Burden Scale (DLB) [31]) of persons of employable age (18–70) without any mental health problems (NN), with mental health problems (M), with work anxiety (A), and both mental health and work anxiety problems (AM) (N = 2131).

Life Domains (Satisfaction Rated 1 Very Negative to 6 Very Positive for Each Domain)	Without Mental Health Problems, Without Work Anxiety (N = 1699) NN	Work Anxiety (N = 191) A	Mental Health Problems (N = 191) M	Work Anxiety and Mental Health Problems (N = 50) AM	All (N = 2131)	Statistics: Overall Differences Between Groups: ANOVA *p*-Value and Effect Size Eta^2^. Significant Differences of Pairwise Comparisons from Bonferroni-Corrected Post Hoc Tests for Mean Scores
Partnership	4.74 (1.33)	4.46 (1.37)	3.78 (1.70)	3.68 (1.66)	4.60 (1.41)	*p* < 0.001, Eta^2^ = 0.049 NNvsA 0.046, NNvsM < 0.001, NNvsAM < 0.001, AvsM < 0.001, AvsAM 0.003
Sexuality	4.67 (1.22)	4.42 (1.32)	3.79 (1.52)	3.08 (1.45)	4.53 (1.31)	*p* < 0.001, Eta^2^ = 0.068 NNvsM < 0.001, NNvsAM < 0.001, AvsM < 0.001, AvsAM < 0.001, MvsAM 0.003
Children	4.80 (1.27)	4.48 (1.42)	4.13 (1.52)	3.80 (1.50)	4.69 (1.34)	*p* < 0.001, Eta^2^ = 0.033 NNvsA 0.013, NNvsM < 0.001, NNvsAM < 0.001, AvsM 0.067, AvsAM 0.011
Parents	4.91 (1.00)	4.65 (1.18)	4.10 (1.36)	3.52 (1.54)	4.78 (1.11)	*p* < 0.001, Eta^2^ = 0.075 NNvsA 0.013, NNvsM < 0.001, NNvsAM < 0.001, AvsM < 0.001, AvsAM < 0.001, MvsAM 0.005
Friends	5.16 (0.73)	5.03 (0.86)	4.66 (1.07)	4.16 (1.02)	5.08 (0.81)	*p* < 0.001, Eta^2^ = 0.062 NNvsM < 0.001, NNvsAM < 0.001, AvsM < 0.001, AvsAM < 0.001, MvsAM < 0.001
Neighbors	4.51 (0.91)	4.46 (1.17)	4.04 (1.14)	3.64 (1.05)	4.44 (0.98)	*p* < 0.001, Eta^2^ = 0.034 NNvsM < 0.001, NNvsAM < 0.001, AvsM < 0.001, AvsAM < 0.001
Colleagues	4.64 (0.87)	4.40 (1.22)	4.16 (1.08)	3.38 (1.21)	4.55 (0.96)	*p* < 0.001, Eta^2^ = 0.075 NNvsA 0.007, NNvsM < 0.001, NNvsAM < 0.001, AvsM 0.076, AvsAM < 0.001, MvsAM < 0.001
Work	4.67 (0.98)	4.32 (1.33)	3.98 (1.24)	3.02 (1.35)	4.54 (1.09)	*p* < 0.001, Eta^2^ = 0.082 NNvsA < 0.001, NNvsM < 0.001, NNvsAM < 0.001, AvsM 0.013, AvsAM < 0.001, MvsAM < 0.001
Leisure Time	5.09 (0.77)	4.99 (0.86)	4.49 (1.22)	3.96 (1.12)	5.00 (0.87)	*p* < 0.001, Eta^2^ = 0.073 NNvsM < 0.001, NNvsAM < 0.001, AvsM < 0.001, AvsAM < 0.001, MvsAM < 0.001
Health	4.88 (0.92)	4.75 (1.08)	3.81 (1.30)	2.92 (1.25)	4.73 (1.07)	*p* < 0.001, Eta^2^ = 0.149 NNvsM < 0.001, NNvsAM < 0.001, AvsM < 0.001, AvsAM < 0.001, MvsAM < 0.001
Finances	4.36 (1.09)	4.15 (1.40)	3.53 (1.48)	2.52 (1.28)	4.22 (1.22)	*p* < 0.001, Eta^2^ = 0.080 NNvsM < 0.001, NNvsAM < 0.001, AvsM < 0.001, AvsAM < 0.001, MvsAM < 0.001
Housing	4.88 (0.92)	4.69 (1.17)	4.13 (1.35)	3.58 (1.31)	4.77 (1.03)	*p* < 0.001, Eta^2^ = 0.076 NNvsM < 0.001, NNvsAM < 0.001, AvsM < 0.001, AvsAM < 0.001, MvsAM 0.003
Environment	3.78 (1.15)	4.25 (1.07)	3.41 (1.18)	3.36 (1.30)	3.78 (1.16)	*p* < 0.001, Eta^2^ = 0.027 NNvsA < 0.001, NNvsM < 0.001, AvsM < 0.001, AvsAM < 0.001
Heimat	4.71 (0.93)	4.69 (1.07)	4.16 (1.22)	3.40 (1.37)	4.63 (1.02)	*p* < 0.001, Eta^2^ = 0.059 NNvsM < 0.001, NNvsAM < 0.001, AvsM < 0.001, AvsAM < 0.001, MvsAM < 0.001
Politics	3.12 (1.14)	3.31 (1.40)	2.65 (1.27)	2.82 (1.32)	3.08 (1.19)	*p* < 0.001, Eta^2^ = 0.017 NNvsM < 0.001, AvsM < 0.001
Future	4.14 (1.15)	4.10 (1.28)	3.35 (1.25)	2.82 (1.12)	4.03 (1.20)	*p* < 0.001, Eta^2^ = 0.059 NNvsM < 0.001, NNvsAM < 0.001, AvsM < 0.001, AvsAM < 0.001, MvsAM 0.026
Life Review	4.60 (0.97)	4.49 (1.08)	3.81 (1.19)	3.22 (1.23)	4.49 (1.05)	*p* < 0.001, Eta^2^ = 0.080 NNvsM < 0.001, NNvsAM < 0.001, AvsM < 0.001, AvsAM < 0.001, MvsAM 0.001

Note: *p*-values and Eta^2^ for overall group comparison are reported in the first line of the Statistics column. In case of significant overall differences in ANOVA, *p*-values of pairwise Bonferroni-corrected post hoc test are shown for continuous variables, e.g., NNvsA 0.046 means there is a significant difference with *p* = 0.046 between the group without mental health or work anxiety problems (NN) and those with work anxiety (A).

**Table 3:** Perceived importance of different life values (Schwartz Short Value Scale [41]) of persons of employable age (18–70) without any mental health problems (NN), with mental health problems (M), with work anxiety (A), and both mental health and work anxiety problems (AM) (N = 2131).

Life Values (Satisfaction Rated 1, Not at All Important, to 6, Very Important)	Without Mental Health Problems, Without Work Anxiety (N = 1699) NN	Work Anxiety (N = 191) A	Mental Health Problems (N = 191) M	Work Anxiety and Mental Health Problems (N = 50) AM	All (N = 2131)	Statistics: Overall Differences Between Groups: ANOVA *p*-Value and Effect Size Eta^2^. Significant Differences of Pairwise Comparisons from Bonferroni-Corrected Post Hoc Tests for Mean Scores
Power	3.29 (1.37)	3.58 (1.40)	2.69 (1.34)	2.50 (1.37)	3.25 (1.39)	*p* < 0.001, Eta^2^ = 0.027 NNvsA 0.042, NNvsM < 0.001, NNvsAM < 0.001, AvsM < 0.001, AvsAM < 0.001
Achievement	3.50 (1.36)	3.56 (1.33)	3.06 (1.35)	2.90 (1.47)	3.45 (1.37)	*p* < 0.001, Eta^2^ = 0.013 NNvsM < 0.001, NNvsAM 0.013, AvsM 0.002, AvsAM 0.014
Hedonism	4.30 (1.06)	4.13 (1.11)	3.94 (1.23)	3.86 (1.45)	4.24 (1.10)	*p* < 0.001, Eta^2^ = 0.013 NNvsM < 0.001, NNvsAM 0.029
Stimulation	3.94 (1.17)	3.88 (1.26)	3.42 (1.33)	3.14 (1.38)	3.87 (1.21)	*p* < 0.001, Eta^2^ = 0.023 NNvsM < 0.001, NNvsAM < 0.001, AvsM < 0.001, AvsAM < 0.001
Self-direction	4.92 (0.98)	4.61 (1.12)	4.67 (1.11)	4.29 (1.19)	4.85 (1.02)	*p* < 0.001, Eta^2^ = 0.019 NNvsA < 0.001, NNvsM 0.008, NNvsAM < 0.001
Universalism	4.84 (0.99)	4.40 (1.18)	4.81 (1.07)	4.52 (1.26)	4.79 (1.03)	*p* < 0.001, Eta^2^ = 0.016 NNvsA < 0.001, AvsM < 0.001
Benevolence	4.91 (0.91)	4.46 (1.15)	4.84 (1.92)	4.36 (1.16)	4.85 (0.95)	*p* < 0.001, Eta^2^ = 0.025 NNvsA < 0.001, NNvsAM < 0.001, AvsM < 0.001, MvsAM 0.008
Tradition	4.29 (1.22)	4.07 (1.30)	3.91 (1.33)	3.56 (1.44)	4.22 (1.25)	*p* < 0.001, Eta^2^ = 0.016 NNvsM < 0.001, NNvsAM < 0.001
Conformity	4.26 (1.13)	4.05 (1.31)	3.99 (1.23)	4.08 (1.32)	4.21 (1.16)	*p* = 0.002, Eta^2^ = 0.007 NNvsM 0.013
Stability	4.74 (0.96)	4.46 (1.04)	4.70 (1.09)	4.18 (1.22)	4.70 (0.99)	*p* < 0.001, Eta^2^ = 0.013 NNvsA < 0.001, NNvsAM < 0.001, MvsAM 0.005

Note: *p*-values and Eta^2^ for overall group comparison are reported in the first line of the Statistics column. In case of significant overall differences in ANOVA, *p*-values of pairwise Bonferroni-corrected post hoc test are shown for continuous variables, e.g., NNvsA 0.042 means there is a significant difference, with *p* = 0.046 between the group without mental health or work anxiety problems (NN) and those with work anxiety (A).

**Table 4:** Job coping expectation (JoCoRi [5]) of persons of employable age (18–70) without any mental health problems (NN), with mental health problems (M), with work anxiety (A), and both mental health and work anxiety problems (AM) (N = 2131).

Job Coping Behavior (Each Item Rated from 0 Not at All to 4 Fully Agree)	Without Mental Health Problems, Without Work Anxiety (N = 1699) NN	Work Anxiety (N = 191) A	Mental Health Problems (N = 191) M	Work Anxiety and Mental Health Problems (N = 50) AM	All (N = 2131)	Statistics: Overall Differences Between Groups: ANOVA *p*-Value and Effect Size Eta^2^. Significant Differences of Pairwise Comparisons from Bonferroni-Corrected Post Hoc Tests for Mean Scores
When I get nervous or stressed at work, I can calm myself down.	3.09 (1.00)	2.81 (1.18)	2.44 (1.23)	1.58 (1.13)	2.97 (1.08)	*p* < 0.001, Eta^2^ = 0.072 NNvsA 0.003, NNvsM < 0.001, NNvsAM < 0.001, AvsM 0.003, AvsAM < 0.001, MvsAM < 0.001
I can tolerate that I do not feel my best at work all the time.	2.78 (1.06)	2.49 (1.18)	2.27 (1.15)	1.82 (1.15)	2.69 (1.10)	*p* < 0.001, Eta^2^ = 0.036 NNvsA 0.002, NNvsM < 0.001, NNvsAM < 0.001, AvsAM < 0.001
When a conflict arises at work, I address it or I help actively to solve the problem.	2.97 (1.00)	2.74 (1.02)	2.41 (1.14)	1.94 (1.15)	2.88 (1.04)	*p* < 0.001, Eta^2^ = 0.045 NNvsA 0.015, NNvsM < 0.001, NNvsAM < 0.001, AvsM 0.012, AvsAM < 0.001, MvsAM 0.021
When I have problems with job assignments or work procedures, I start searching for information or turn to the person in charge.	3.05 (0.96)	2.63 (1.06)	2.69 (1.12)	2.04 (1.19)	2.96 (1.01)	*p* < 0.001, Eta^2^ = 0.042 NNvsA < 0.001, NNvsM < 0.001, NNvsAM < 0.001, AvsAM 0.001, MvsAM < 0.001
When I have too much work, I say to myself that I will manage this and I begin with a first step.	3.12 (0.91)	2.69 (0.98)	2.68 (1.07)	2.10 (1.15)	3.02 (0.97)	*p* < 0.001, Eta^2^ = 0.052 NNvsA < 0.001, NNvsM < 0.001, NNvsAM < 0.001, AvsAM < 0.001, MvsAM < 0.001
I can work together with colleagues and supervisors as well as with those whom I do not like personally.	3.00 (0.95)	2.67 (1.06)	2.60 (1.19)	1.94 (1.11)	2.91 (1.00)	*p* < 0.001, Eta^2^ = 0.043 NNvsA < 0.001, NNvsM < 0.001, NNvsAM < 0.001, AvsAM < 0.001, MvsAM < 0.001
When I am impaired at work due to health problems, I tell this to my superior in a way that helps him understand the problem so that we can search for a solution together.	2.83 (1.06)	2.75 (1.02)	2.39 (1.24)	2.16 (1.11)	2.77 (1.09)	*p* < 0.001, Eta^2^ = 0.021 NNvsM < 0.001, NNvsAM < 0.001, AvsM 0.007, AvsAM < 0.001

Note: *p*-values and Eta^2^ for overall group comparison are reported in the first line of the Statistics column. In case of significant overall differences in ANOVA, *p*-values of pairwise Bonferroni-corrected post hoc test are shown for continuous variables, e.g., NNvsA 0.002 means there is a significant difference with *p* = 0.002 between the group without mental health or work anxiety problems (NN) and those with work anxiety (A).

**Table 5:** Intentional Recreational Activities of Daily Life (RADL scale [32]) of persons of employable age (18–70) without any mental health problems (NN), with mental health problems (M), with work anxiety (A), and both mental health and work anxiety problems (AM) (N = 2131). Also included is the percentage of persons who perform the respective activities regularly with the intention of feeling better.

Intentional Recreative Activities of Daily Living	Without Mental Health Problems, Without Work Anxiety (N = 1699) NN	Work Anxiety (N = 191) A	Mental Health Problems (N = 191) M	Work Anxiety and Mental Health Problems (N = 50) AM	All (N = 2131)	Statistics: Overall Differences Between Groups Chi^2^ in Frequencies, *p*-Value
Social encounters	24.8%	27.9%	23.0%	18.0%	24.8%	0.469
Television, internet	17.8%	30.0%	25.7%	40.0%	20.1%	<0.001
Relaxation and silence	9.8%	16.4%	14.1%	24.0%	11.1%	<0.001
Cooking and eating	13.2%	13.2%	16.8%	18.0%	13.6%	0.421
Excursions, traveling, and ventures	7.8%	10.1%	6.3%	10.0%	7.9%	0.530
Listening to and making music	12.5%	17.4%	16.8%	16.0%	13.4%	0.115
Hobbies	8.4%	15.9%	9.6%	16.0%	9.3%	0.003
Wellness	6.6%	6.9%	7.3%	12.2%	6.8%	0.469
Reading and literature	7.4%	9.5%	7.9%	10.0%	7.7%	0.691
Sports and exercise	13.0%	10.6%	13.2%	6.0%	12.6%	0.397
Activities in nature	8.0%	11.1%	10.5%	10.0%	8.5%	0.343
Enjoying culture	3.9%	4.7%	4.7%	2.0%	4.0%	0.780

Note: Overall *p*-values are reported, e.g., using relaxation and silence to feel better is reported (*p* < 0.001), with different frequencies for the four groups. Enjoying culture was reported (*p* = 0.780) at similar frequencies in the four groups.

## Data Availability

Data are available from the author upon request.
